# Postmortem succession of gut microbial communities in deceased human subjects

**DOI:** 10.7717/peerj.3437

**Published:** 2017-06-12

**Authors:** Jennifer M. DeBruyn, Kathleen A. Hauther

**Affiliations:** 1 Biosystems Engineering & Soil Science, University of Tennessee Institute of Agriculture, Knoxville, TN, United States of America; 2 Department of Anthropology, University of Tennessee—Knoxville, Knoxville, TN, United States of America

**Keywords:** Microbiome, Postmortem interval, Microbial ecology, Human decomposition, Anthropology research facility, Forensic anthropology

## Abstract

The human microbiome has demonstrated an importance for the health and functioning in living individuals. However, the fate of the microbiome after death is less understood. In addition to a better understanding of microbe-mediated decomposition processes, postmortem succession of human-associated microbial communities has been suggested as a possible forensic tool for estimating time since death, or postmortem interval (PMI). The objective of our study was to document postmortem changes in human gut bacterial communities. Gut microflora were repeatedly sampled from the caeca of cadavers as they decayed under natural environmental conditions. 16S rRNA gene amplicon sequencing revealed that over time, bacterial richness significantly increased (*r*_*s*_ = 0.449) while diversity decreased (*r*_*s*_ =  − 0.701). The composition of gut bacterial communities changed in a similar manner over time towards a common decay community. OTUs belonging to Bacteroidales (*Bacteroides*, *Parabacteroides*) significantly declined while Clostridiales (*Clostridium*, *Anaerosphaera*) and the fly-associated Gammaproteobacteria *Ignatzschineria* and *Wohlfahrtiimonas* increased. Our examination of human caeca microflora in decomposing cadavers adds to the growing literature on postmortem microbial communities, which will ultimately contribute to a better understanding of decomposition processes.

## Introduction

Decomposition of vertebrate mortalities is driven in part by microbial activity. Following death, a lack of oxygen in the body results in cell autolysis, releasing macromolecules. The body’s resident microbes, particularly those concentrated in the gastrointestinal tract, metabolize these cellular products in the process of putrefaction. The fermentative activities of these microbes cause bloating as gasses build up inside the carcass, and ultimately liquefaction of tissues (Carter, Yellowlees & Tibbett, 2007). While it is well accepted the human microbiome plays an important role in tissue decomposition, the composition and dynamics of these microbial communities postmortem are only starting to receive attention.

Understanding the processes and dynamics of decomposition has application in forensic science, particularly with respect to developing robust estimates of time since death, or postmortem interval (PMI). All methods have limitations, thus using a combination of approaches typically provides the best estimates of PMI (Mathur & Agrawal, 2011). Therefore, there is an ongoing need to develop and validate new PMI estimation methods. There has been recent interest in the use of microbial communities in the decomposition environment as markers of PMI: if the postmortem succession of microbial communities is repeatable and predictable, then it may be possible to use the communities as forensic indicators, similar to the approach taken by forensic entomology (Amendt, Krettek & Zehner, 2004). Recent studies have begun describing human postmortem microbial communities associated with a variety of habitats, including skin (Metcalf et al., 2016; Pechal et al., 2017; Hyde et al., 2015), mouth and rectum (Hyde et al., 2015; Hyde et al., 2013), ear and nasal canals (Johnson et al., 2016), internal organs (Javan et al., 2016; Tuomisto et al., 2013), bones (Damann, Williams & Layton, 2015) and soils below (Metcalf et al., 2016; Cobaugh, Schaeffer & DeBruyn, 2015). Other studies have also reported postmortem changes associated with decomposing animal carcasses (Pechal et al., 2013a; Metcalf et al., 2013; Heimesaat et al., 2012; Burcham et al., 2016; Dickson et al., 2011). Collectively these studies are beginning to reveal general patterns of microbial succession, which include a shift towards a higher relative abundance of anaerobic taxa. Attempts to relate taxa abundances or community patterns to PMI have demonstrated that bacterial community composition may be able to predict PMI with an accuracy of a few days (Metcalf et al., 2016; Johnson et al., 2016).

Here we expanded on this body of work to include bacterial communities of the proximal large intestine (caecum); a habitat not previously investigated using high throughput sequencing (to our knowledge). In a previous study, we sampled gut microflora of the caeca of six deceased human subjects repeatedly following death, until tissues were too compromised to distinguish (Hauther et al., 2015). Using qPCR, we found populations of *Bacteroides* and *Lactobacillus* declined exponentially as decay progressed. This suggested that the microbial communities changed in structure over time, and led to the hypothesis that other populations of bacteria may be useful as biomarkers of time since death. Our previous study was limited to examining only three microbial populations using targeted qPCR. Therefore, the objective of this study was to characterize and quantify the entire bacterial community of the human gut following death as decomposition progresses. We hypothesized the communities would change in structure with time, with a decline in Bacteroidetes, and increase in more robust taxa such as Clostridia and Proteobacteria. The changes in intestinal microflora have been examined in mice up to 48 h postmortem (Metcalf et al., 2013; Heimesaat et al., 2012). Here we present the postmortem changes in gut microflora of humans, up to 30 days following death.

## Materials and Methods

### Human decomposition and sampling

Four deceased human subjects were placed at the University of Tennessee, Knoxville, Anthropology Research Facility (ARF) in the summer of 2011, as part of a larger study described previously (Hauther et al., 2015). The individuals were donations to the University of Tennessee, Knoxville, Forensic Anthropology Center (FAC) for the W. M. Bass Donated Skeletal Collection (http://web.utk.edu/ fac/collection.html). As no living human subjects were involved, this work was exempt from review by the University of Tennessee Institutional Review Board. No preference was employed for sex, age, ancestry, weight, etc. FAC standard protocol for accepting donors ensured the individuals did not have communicable diseases. The bodies were not autopsied or embalmed; they were immediately refrigerated after death and placed at the ARF within three days. The four individuals (referred to as A6, B6, C5, D6) ranged in age at death from 62 to 67 years and weights from 56 to 77 kg, and all died of natural causes. A small incision was made in the abdomen and sterile swabs were used to collect gut material from the caecum. The incision was sealed with tape and re-sampled daily until tissues were too decayed to identify. Sampling times were converted to cumulative degree hours (CDH) based on hourly measurements of air temperature from a local meteorological station as previously described (Hauther et al., 2015). Since all individuals were decomposing at the same time in relatively close proximity, insect activity was comparable between them. While a comprehensive survey of arthropod communities was not included as a part of this study, we did observe extensive activity by Calliphoridae larvae, as has been previously documented at this facility (Schoenly et al., 2007).

### DNA sequencing and amplicon library analyses

DNA was extracted using a MoBio PowerSoil DNA extraction kit. 16S rRNA gene libraries (V4 region) were built and sequenced on the Illumina MiSeq platform at the Hudson Alpha Genomic Service Lab (Huntsville, AL, USA) using primers and conditions described in Caporaso et al. (2012). Sequence QC and analysis was done in Mothur v.1.37.0 (Schloss et al., 2009) as previously described (Cobaugh, Schaeffer & DeBruyn, 2015). Briefly, forward and reverse reads were joined; then, sequences containing ambiguous bases, homopolyers longer than eight nucleotides, and unreasonable amplicon lengths (<248 or >275 bp) were removed. Sequences were aligned to the Silva reference alignment, preclustered, and screened for chimeras using UChime. Sequences that were taxonomically classified as something other than bacteria (i.e., chloroplasts, mitochondria, Eukaryota, or Archaea) were removed. OTUs were determined based on phylotype; sequences were taxonomically classified against the RDP database (>80% similarity), classified by genus, and clustered according to taxonomy. Across all samples, 734 unique OTUs were identified. Raw sequences were deposited in the NCBI Short Read Archive (SRP098575).

Prior to alpha-diversity calculations, libraries were subsampled to 25,082 reads per library (based on the smallest library). Alpha diversity statistics were calculated in Mothur v.1.37.0, and included Good’s coverage estimate, richness (number of OTUs), Simpson’s Diversity index. Beta diversity analyses were done in Primer v6 (Clarke & Gorley, 2006). OTU counts were first standardized by samples to yield relative abundances, then square root transformed to down weight highly abundant species. Bray–Curtis distances between samples were calculated. Hierarchical group average clustering of Bray–Curtis distances was done in Primer v6 to identify clusters of samples. There was an obvious clustering at 37% similarity; these clusters were then assessed for significant differences in multivariate structure using a PERMANOVA analysis with unrestricted permutation of raw data. Differences in alpha diversity metrics between the two clusters were assessed using Student’s *T* test. Nonmetric multidimensional scaling on Bray–Curtis distances was performed to visualize community similarities and differences.

We additionally used the Similarity Percentages (SIMPER) routine in Primer, using a one way design on the calculated Bray–Curtis similarities, to determine the contribution of each OTU to the dissimilarity between early and late groups and identify the taxa that contribute the most to the observed patterns (Clarke & Gorley, 2006). Non-parametric Spearman’s rank correlation coefficients (*r*_*s*_) between the top 30 most abundant OTUs (with relative abundances >  0.3) and postmortem time were calculated in *R* with a Bonferroni adjustment for multiple comparisons (adjusted α < 0.0016) (R Core Team, 2013).

### Influence of sampling

The sampling method, which involved introduction of a sterile swab into the abdominal cavity, undoubtedly introduced some oxygen to the gut, which would have normally been anaerobic. We attempted to limit this introduction as much as possible and included several bodies as controls to determine if the sampling had an effect. The method and controls are discussed in our previous study (Hauther et al., 2015). The six control bodies were only sampled once at various PMIs, and analyzed along with six test bodies. We found no difference in population relative abundances between control and test bodies for all but the rare populations (results provided in Hauther et al., 2015). Thus, based on our research questions, we concluded that our sampling method did not have a substantial effect on the dominant gut bacterial populations.

## Results

**Figure 1:**
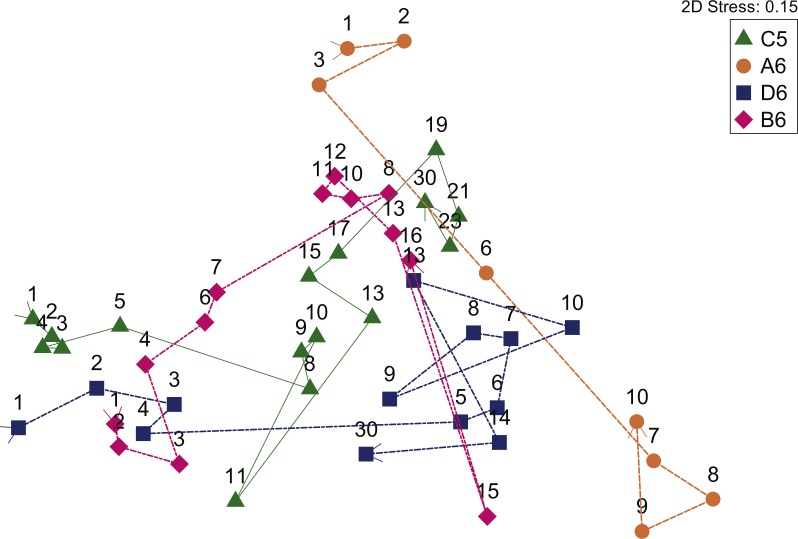
NMDS of Bray–Curtis distances revealed the changes in deceased human subjects gut bacterial communities over time. Symbols represent the three individuals: A6 (circles), B6 (diamonds), C5 (triangles), D6 (squares) and numbers refer to the day postmortem. The lines indicate the trajectory of the communities starting from the first day postmortem.

After processing to remove erroneous sequences, 8,756,105 sequences remained in our dataset. The sample with the least number of sequences contained 25,082, so all other samples were randomly subsampled to this size. At 25,082 sequences per sample, mean Good’s coverage for all samples was 0.998. Replicate libraries were made for a random selection of four samples to assess technical error. Duplicates were more similar to each other than to other libraries, with Bray–Curtis similarities of community composition ranging from 92.06 to 93.52 at the OTU level and 97.55 to 98.77 at the phylum level. Initial screening of the sequence libraries revealed that one individual (A6), which had a percutaneous endoscopic gastrostomy (feeding) tube prior to death, had very different starting postmortem gut microbial communities compared to the other three (Fig. 1). The communities in A6 did change over time, but because these communities were distinct from the other three individuals, the inter-individual differences would obscure any time-based differences in the other three. Thus, we did not include A6 in the alpha diversity, SIMPER or correlation analyses.

**Figure 2:**
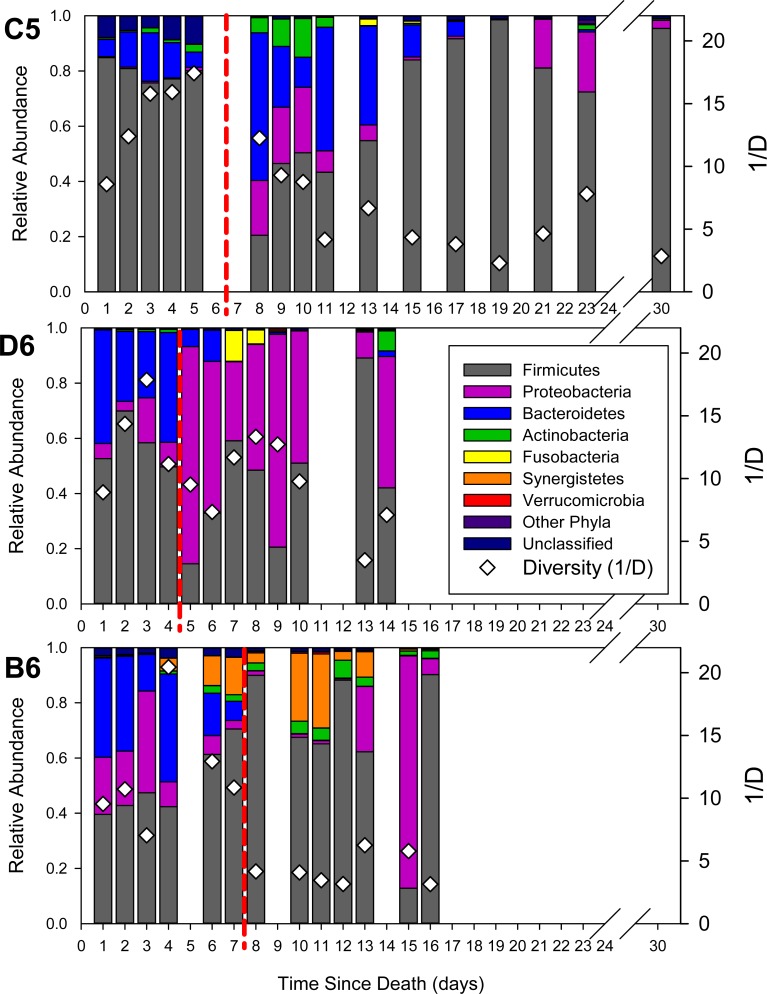
Relative abundance of phyla in the bacterial communities as a function of time since death (postmortem interval). Red dashed lines differentiate the ‘early’ from the ‘late’ communities as determined by hierarchical cluster analysis. White diamonds show the community diversity, as estimated by the inverse Simpson’s Index (1/D).

Over time, the taxon richness of the bacterial communities increased, while the diversity significantly decreased, indicating a decrease in evenness (Fig. 2 and Fig. S1). The number of OTUs was significantly correlated to time since death (Spearman’s rho (*r*_*s*_) = 0.449, *p* = 0.003). In contrast, diversity (Simpson’s Index) was significantly inversely correlated to time (*r*_*s*_ =  − 0.701, *p* < 0.001) (Fig. S1). An NMDS analysis of Bray–Curtis similarities revealed that the microbial communities in these bodies changed over time (Fig. 1). Over the course of the study, the mean similarities of the communities within each body were low (44.74–50.31%). This analysis revealed that while there was considerable variation between individuals, there was a change along a similar trajectory for all three. A hierarchical group average clustering of communities revealed a distinct split in the communities (at 37% similarity). This split was indicative of a time-depended shift, with one cluster comprised of samples taken earlier in decay, and the other of samples taken later on (Fig. 1 and Fig. S2). This shift from ‘early’ to ‘late’ happened in the middle of the traditionally defined ‘bloat’ stage (*sensu* Payne, 1965) around the same time for the three individuals: after days five, four, and seven for C5, D6, and B6, respectively. When corrected for environmental temperature differences, these time periods represent cumulative degree hours (CDH) 77–173, 67–91, 155–184, respectively. The ‘early’ and ‘late’ communities were significantly different in structure (PERMANOVA Pseudo-*F* = 19.974, *p* = 0.001).

The ‘early’ microbial communities had a mean of 135 ± 17 OTUs. The diversity was high, with a mean inverse Simpson’s Index of 12.93 ± 3.91. They had an average Bray–Curtis similarity of 59.42%. Major phyla in these communities were Firmicutes and Bacteroidetes,characteristic of human gut communities (Fig. 2). A SIMPER analysis was used to determine which OTUs contributed most to the difference between early and late microbial communities (Table S1). This analysis revealed early communities had significantly higher abundances of *Bacteroides* and *Parabacteroides* (Phylum: Bacteroidetes), and the Firmicutes *Faecalibacterium*, *Phascolarctobacterium*, *Blautia*, *Lachnospiraceae incertae sedis*.

The ‘late’ microbial communities had a higher richness but lower diversity than the early microbial communities: the late communities had a mean of 186 ± 78 OTUs, which was significantly higher than the early communities (two-tailed *T* test, *t* =  − 3.215, *p* = 0.003). These late microbial communities were also more variable compared to the early microbial communities, with a mean Bray–Curtis similarity of 49.45%. The inverse of the Simpson’s Diversity Index was 6.608 ± 3.78, indicating significantly lower diversity than the early communities (*t* = 5.232, *p* < 0.001). Firmicutes still dominated, but we observed reduced relative abundances of Bacteroidetes at the phylum level (Fig. 2). These communities were significantly enriched in OTUs belonging to order Clostridiales within phylum Firmicutes (*Clostridium, Peptostreptococcus,* and *Anaerosphaera*), and Gammaproteobacteria OTUs (*Wohlfahrtiimonas*, *Ignatzschineria*, *Acinetobacter* and *Providencia*) (Table S1).

**Figure 3:**
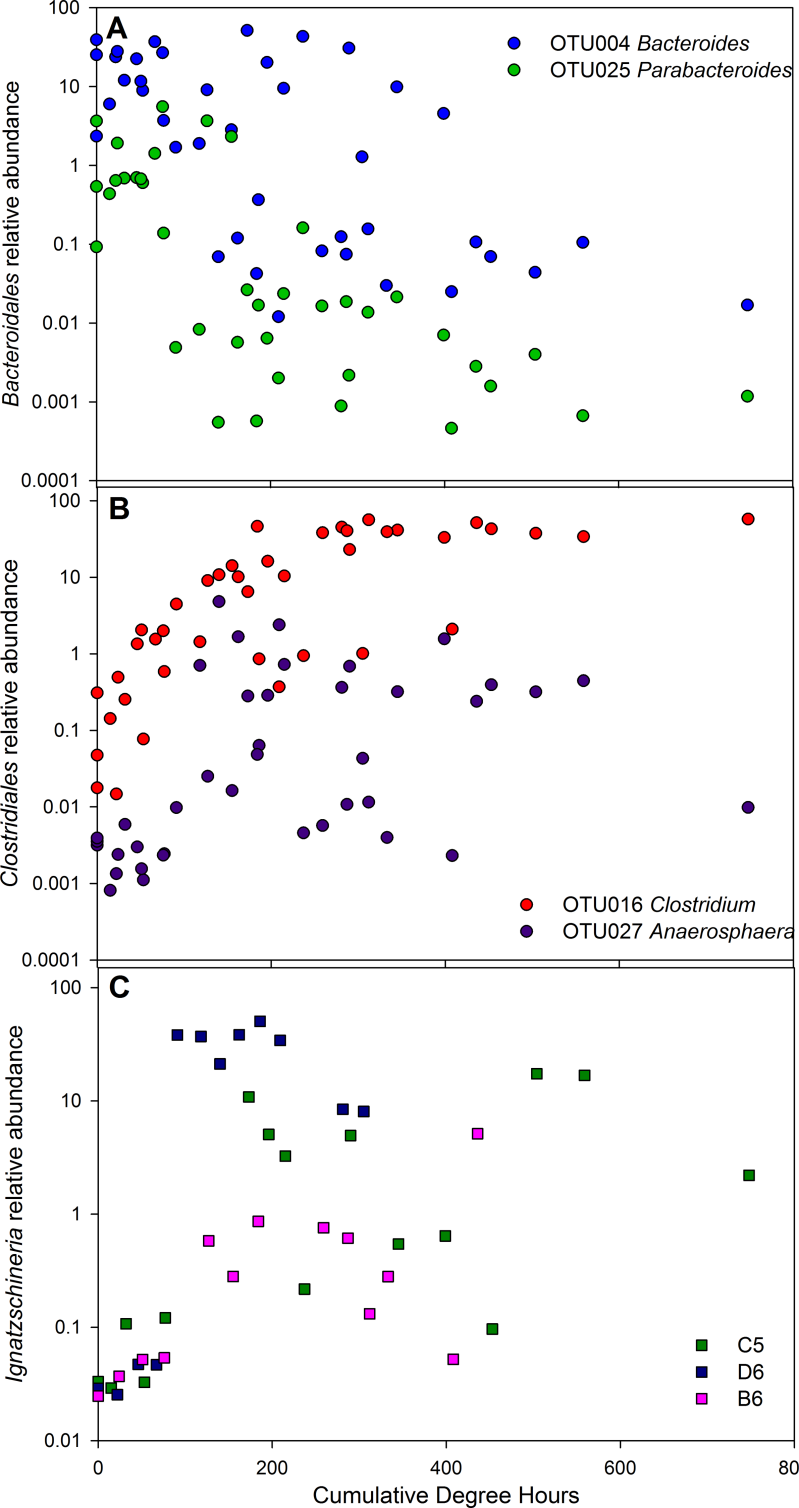
Relative abundance of OTUs as a function of time since death (cumulative degree hours). (A) Bacteroidales genera *Bacteroides* and *Parabacteroides*; (B) Clostridiales genera *Clostridium* and *Anaerosphaera*; (C) Gammaproteobacteria *Ignatzschineria* for each of the three individuals showing the inter-individual variability for this taxon.

While there was a clear change in the postmortem gut microbial community with time, there were some differences between the three individuals. For example, we observed increases in Proteobacteria in two of the individuals, and an increase in Synergistetes in the third (Fig. 2). At the OTU level, we observed significant secondary clustering within the ‘late’ cluster (Fig. S2): the later C5 and B6 samples clustered together while D6 had slightly different community structures (PERMANOVA Pseudo *F* = 6.992, *p* = 0.001), largely due to the proliferation of Proteobacteria and increase in *Ignatzschineria* (Fig. 3C).

To determine the utility of using individual OTUs as a predictor of time since death, or postmortem interval (PMI), we examined the relationships between OTUs and CDH. Table 1 lists OTUs with a significant correlation to CDH, positive or negative. The strongest individual predictors of PMI were two Bacteroidales OTUs (Fig. 3A) which declined with time and two Clostridiales OTUs that increased over time (Fig. 3B).

**Table 1:** Pearson’s correlation coefficients (*r*) between log transformed OTU relative abundance and postmortem interval (as cumulative degree hours, CDH) for OTUs that were significantly correlated (*p* < 0.0016) to CDH.

OTU#	Phylum	Order	Family	Genus	*r*
4	Bacteroidetes	Bacteroidales	Bacteroidaceae	*Bacteroides*	−0.635
25			Porphyromonadaceae	*Parabacteroides*	−0.627
16	Firmicutes	Clostridiales	Clostridiacea	*Clostridium*	0.717
27			Incertae_Sedis_XI	*Anaerosphaera*	0.417
26			Lachnospiraceae	*Blautia*	−0.600
12			Ruminococcaceae	*Faecalibacterium*	−0.607
20		Lactobacillales	Enterococcaceae	Unclassified	0.706
3	Proteobacteria (Gamma)	Xanthomonadales	Xanthomonadaceae	*Ignatzschineria*	0.406

## Discussion

In this study we documented the shifts in human gut microflora postmortem, as deceased human subjects decayed in an outdoor environment. While other studies of postmortem human microbial communities have included fecal or rectal sampling (e.g., Hyde et al., 2015; Hyde et al., 2013), here we provide an examination of communities in the proximal large intestine (caecum). The bacterial communities in these decomposing individuals had a distinct compositional shift in the middle of the bloat phase of decomposition, between days four and seven in this study. Prior to this point in decomposition, the communities were relatively diverse and similar between three individuals, with bacterial assemblages appearing typical of human gut communities (The Human Microbiome Consortium, 2012). Mid-way through bloat, the communities underwent a distinct shift. The timing of this shift may correspond to a change in the physical environment (e.g., liquefaction of surrounding cells and structures), chemical environment (e.g., buildup of putrefaction byproducts) and/or biological competition (e.g., sensitive members of the human gut flora die off while more resilient members proliferate; and/or invasion of external microbes).

Over time, the bacterial communities exhibited decreased diversity. As richness was not affected, this indicates a reduction of evenness, as the communities become dominated by a few dominant genera. This is typical of a disturbance response or a bloom event. In a decomposing body, likely both scenarios occurred: the rapidly shifting environmental conditions and production of putrefaction products likely stressed some members of the community, while others were more tolerant and proliferated on the newly available substrates. It was notable the variation between individuals increased in the late communities; that is, bacterial communities were more similar between the three individuals in the beginning. External factors such as temperature, soils, and arthropod communities were relatively similar between the individuals, as they were all placed in the same environment around the same time. However, internal differences that we could not control for (e.g., body fat content, presence of drugs or drug metabolites, etc.) may have influenced the trajectory of the decomposer communities. For example, individual A6 had a remarkably different starting microbiome, and exhibited an altered decomposition trajectory from the other three (though interestingly, this subject also exhibited a clear shift during decomposition). It should also be recognized that decomposition, because of its highly dynamic nature, is not entirely deterministic and would be subject to stochasticity that would result in slightly different trajectories of the bacterial communities between individuals. Other studies of microbial community succession have shown that the relative importance of stochastic and deterministic (i.e., environmental selection) processes is generally time dependent, with stochastic processes dominating early in succession, and deterministic processes becoming more important later on (Dini-Andreote et al., 2015; Ferrenberg et al., 2013).

Despite the inter-individual variation, we documented several patterns consistent between all three individuals. The changes from ‘early’ to ‘late’ communities were driven in part by pronounced changes in OTUs classifying as Clostridiales. *Clostridium* spp. are normal members of the human gut microflora, and have been reported as a prominent members of postmortem microbial communities, including both internal organs and external sites (Hyde et al., 2015; Javan et al., 2016; Tuomisto et al., 2013; Cobaugh, Schaeffer & DeBruyn, 2015; Metcalf et al., 2013; Can et al., 2014). Another Clostridiales, *Anaerosphaera* spp., have been previously isolated from animal waste reactors and identified as aminolytic anaerobes, fermenting amino acids into volatile fatty acids (Ueki et al., 2009), implicating them as members of the putrefactive consortia as well. Mouse model studies examining translocation of bacteria postmortem have demonstrated the migration and proliferation of *Clostridium* and other anaerobic taxa in the internal organs with increasing postmortem intervals (Heimesaat et al., 2012; Burcham et al., 2016); an increase in *Clostridium* in human internal organs postmortem has also been reported (Javan et al., 2016; Tuomisto et al., 2013). Clostridia are known putrefactive organisms, so their observed increase in relative abundance may be due to an increase in vegetative growth, where they gained energy through the fermentation of cellular products. Alternatively, but less likely, their ability to form endospores may have allowed them to withstand the stressful conditions within the decomposing body, while other taxa were reduced in abundance.

The other groups significantly enriched in the late communities included several Gammaproteobaccteria. The increase in *Ignatzschineria* and to a lesser extent, *Wohlfahrtiimonas*, was intriguing; these taxa and have been identified in other studies of postmortem bacterial communities (Hyde et al., 2015; Hyde et al., 2013; Cobaugh, Schaeffer & DeBruyn, 2015). *Ignatzschineria* spp. have been identified in flesh flies (Diptera: Sarcophagidae) (Gupta et al., 2011; Le Brun et al., 2015; Gupta et al., 2014) and blow flies (Diptera: Calliphoridae) (Singh et al., 2015). *Wohlfahrtiimonas* spp. have also been associated with fly larvae (Gupta et al., 2014; Campisi, Mahobia & Clayton, 2015; Lee et al., 2014). Thus, it is possible that flies visiting the bodies introduced these bacteria. Their dominance in the overall bacterial community and significant increase in relative abundance over the postmortem interval (PMI) suggests they are either introduced in large quantities by the extensive insect activity and/or proliferate in this environment. Previous work has indicated that insects on carrion may alter microbial community structure and functioning (Pechal et al., 2013b); this raises important questions about the possible role of insect-introduced bacteria in decomposition.

There has been increasing interest in determining if patterns in postmortem microbial communities may be useful in a forensic context. To this end, we determined the taxa most correlated to time since death, or postmortem interval (PMI). While our sample size is very small, these analyses revealed taxa related to PMI. These taxa include *Bacteroides* and *Parabacteroides*, which declined over time and were significantly inversely correlated to PMI. This corroborates our previous study with six individuals, in which we quantified *Bacteroides* using qPCR and demonstrated a significant, quantifiable decay relationship in the relative abundances of these organisms with increasing PMI (Hauther et al., 2015). *Clostridium* was the strongest positive predictor of PMI.

Our examination of human gut microflora of the caecum in decomposing human subjects adds to the growing literature on postmortem microbial communities. While the small sample size used here limits the interpretation, and would need to be validated by further study, it has provided new insight into postmortem patterns of microbial succession, and thus may contribute to our overall understanding of decomposition processes.

##  Supplemental Information

10.7717/peerj.3437/supp-1 Supplemental Information 1 Supplementary table and figures

10.7717/peerj.3437/supp-2 Supplemental Information 2 Postmortem gut Mothur code for sequence processing

## Additional Information and Declarations

### DNA Deposition

The following information was supplied regarding the deposition of DNA sequences: The sequence data are accessible via the NCBI Short Read Archive SRP#098575: https://www.ncbi.nlm.nih.gov/sra/?term=SRP098575.

### Data Availability

The following information was supplied regarding data availability: The code has been supplied as a Supplementary File.
