# Supplementary material for: Postmortem succession of gut microbial communities in deceased human subjects
Source: PeerJ. 2017 Jun 12;5:e3437. doi: 10.7717/peerj.3437 (PMC5470579; doi:10.7717/peerj.3437)
Supplement: Supplemental Information 1 [file peerj-05-3437-s001.docx]

**Supporting Information**

**Table S1.** Results of a SIMPER analysis, revealing the OTUs that differentiated the early communities from the late communities, contributing at least 1% to the observed dissimilarity.

| OTU | Phylum | Order | Genus | Average Dissimilarity | % Contribution |
| --- | --- | --- | --- | --- | --- |
| *Early* |  |  |  |  |  |
| Otu004 | Bacteroidetes | Bacteroidales | Bacteroides | 2.96 | 4.36 |
| Otu012 | Firmicutes | Clostridiales | Faecalibacterium | 2.07 | 3.04 |
| Otu026 | Firmicutes | Clostridiales | Blautia | 1.37 | 2.02 |
| Otu069 | Firmicutes | Clostridiales | Lachnospiracea incertae sedis | 1.03 | 1.52 |
| Otu025 | Bacteroidetes | Bacteroidales | Parabacteroides | 0.96 | 1.42 |
| Otu048 | Firmicutes | Clostridiales | Oscillibacter | 0.91 | 1.34 |
| Otu042 | Firmicutes | Selenomonadales | Megasphaera | 0.95 | 1.4 |
| Otu067 | Firmicutes | Clostridiales | Ruminococcus | 0.86 | 1.27 |
| Otu062 | Firmicutes | Clostridiales | Clostridium XlVa | 0.82 | 1.21 |
| Otu030 | Firmicutes | Selenomonadales | Acidaminococcus | 0.81 | 1.2 |
| Otu071 | Firmicutes | Clostridiales | Clostridium IV | 0.71 | 1.05 |
| Otu017 | Firmicutes | Selenomonadales | Phascolarctobacterium | 1.2 | 1.77 |
| Otu019 | Proteobacteria | Enterobacteriales | Proteus | 0.94 | 1.39 |
| Otu013 | Synergistetes | Synergistales | Cloacibacillus | 1.06 | 1.56 |
| *Late* |  |  |  |  |  |
| Otu016 | Firmicutes | Clostridiales | Clostridium sensu stricto | 3.5 | 5.15 |
| Otu003 | Proteobacteria | Xanthomonadales | Ignatzschineria | 2.32 | 3.42 |
| Otu015 | Proteobacteria | Xanthomonadales | Wohlfahrtiimonas | 0.84 | 1.24 |
| Otu018 | Proteobacteria | Pseudomonadales | Acinetobacter | 0.72 | 1.06 |


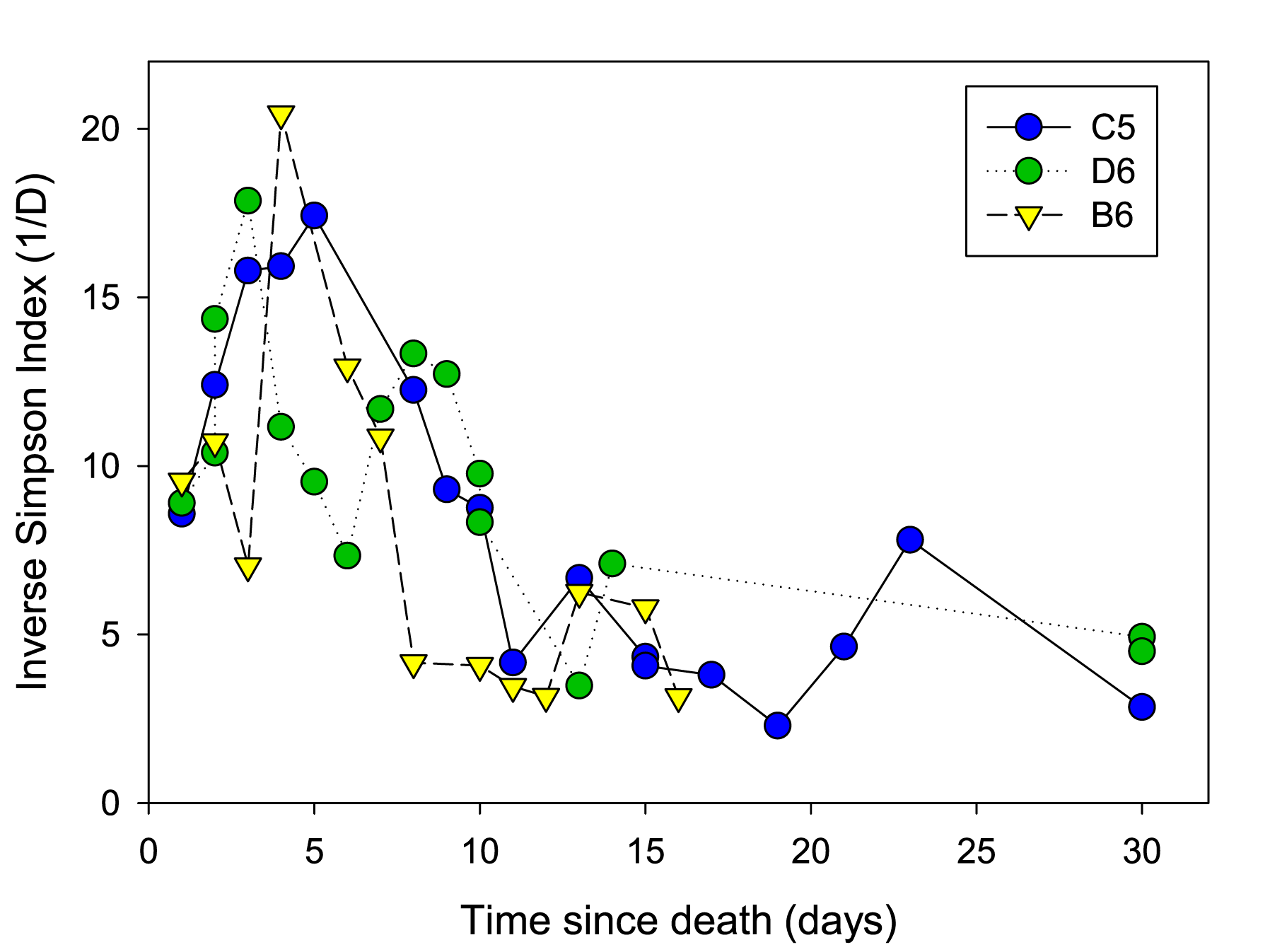


**Figure S1.** Diversity (Inverse Simpson Index) of the gut bacterial communities in three cadavers.


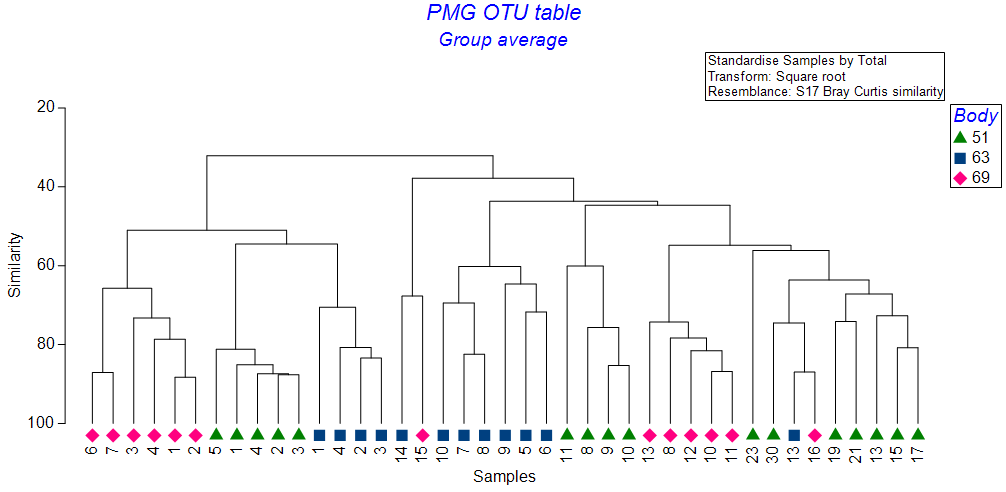


**Figure S2.** Hierarchical group average clustering of Bray-Curtis similarities shows the samples split into two clusters, one corresponding to samples taken early in decay (prior to day 7) and the other to samples taken later in decay. Symbols represent the three individuals: B6 (diamonds), C5 (triangles), D6 (squares) and numbers refer to the day postmortem.
